# Student nurses perceptions on hand hygiene: analysis of behavioral and environmental determinants

**DOI:** 10.1186/1753-6561-5-S6-P279

**Published:** 2011-06-29

**Authors:** D De Wandel, S Labeau, W De Keyzer, D Vogelaers, S Blot

**Affiliations:** 1 Faculty of Health care, University College Ghent, Ghent, Belgium; 2 Faculty of Medicine and Health Sciences, Ghent University, Ghent, Belgium; 3 Department of General Internal Medicine and Infectious Diseases, Ghent University Hospital, Ghent, Belgium

## Introduction / objectives

Hand hygiene compliance is poor among nurses. Hand hygiene compliance is influenced by education. Student nurses’ compliance in clinical practice and perceptions towards hand hygiene have seldom been described.

## Methods

We developed a questionnaire containing demographic data items (i.e. gender and year of education) and items to measure self-reported compliance (10 items, based on the latest WHO recommendations), attitude towards hand hygiene (7 items), perceived social influence (7 items) and observed environmental conditions during clinical practice (5 items).

## Results

The questionnaire was filled out by 181 student nurses of the Faculty of Health Care Vesalius, University College Ghent. Students reported a compliance of 50% or below in the following situations: ‘before and after direct patient contact’ (28%), ‘after glove removal’ (29%), ‘after touching patient surroundings’ (65%). Students reported a positive attitude which remained at the same level during the three years. Social influence increased although not significantly (Kruskal-Wallis P=0.388). The presence of an observer or mentor during clinical practice was reported to increase social pressure. In general, students were satisfied with the environmental conditions (97%) and with the amount of attention the wards spent on hand hygiene (86%). However, students reported insufficient disinfectant on the carts (21%) and in the patient’s rooms (31%).

## Conclusion

Undergraduate student nurses reported poor hand hygiene compliance in several specific situations. The students’ attitude towards hand hygiene was positive. Being observed during clinical practice increases social pressure. Some shortcomings in relation to the ward environment were identified.

## Disclosure of interest

None declared.

